# The influence of men’s masculine gender-role attitude and behaviour on sexual relationships and reproductive health in Malaysia: A cross-sectional study

**DOI:** 10.18502/ijrm.v19i7.9477

**Published:** 2021-08-16

**Authors:** Noraida Endut, Reyhaneh Bagheri, Azman Azwan Azmawati, Intan Hashimah Mohd Hashim, Nor Hafizah Selamat, Leila Mohajer

**Affiliations:** ^1^Centre for Research on Women and Gender (KANITA), Universiti Sains Malaysia, Pulau Pinang, Malaysia.; ^2^School of Communication, Universiti Sains Malaysia, Pulau Pinang, Malaysia.; ^3^School of Social Sciences, Universiti Sains Malaysia, Pulau Pinang, Malaysia.

**Keywords:** Attitudes, Masculinity, Gender-role, Sexual health, Reproductive health, Malaysia.

## Abstract

**Background:**

This article is an extension of a previous research on masculinity and sexual and reproductive health using a newly developed local masculinity scale, KANITA Masculinity Scale, to study men's masculine gender-role attitude and behavior in Malaysia.

**Objective:**

To explore how Malaysian men's masculine gender-role attitude and behavior influence sexual relationships and reproductive health.

**Materials and Methods:**

This cross-sectional study used the localized gender-equitable men scale to measure the attitude of Malaysian men toward sexual relationships and reproductive health. A questionnaire survey was administered to a sample of 168 men of ages 20–64 yr, in Malaysia. Data were analyzed using SmartPLS version 3.2.6.

**Results:**

The findings of the study showed that men's traditional behavior and controlling nature are positively associated with the inequality in sexual relationships (p < 0.05, t = 1.838; and p < 0.05, t = 3.750, respectively) and reproductive health (p < 0.05, t = 2.196; and p < 0.05, t = 4.133, respectively). In other words, men who offer stronger endorsement of traditional behavior and control over women report more negative condom attitude and greater priority of men's desire in sexual relationships. In contrast, there was no significant relationship between men's response to family and feminine roles in men with sexual relationships and reproductive health.

**Conclusion:**

Our findings highlight the importance of considering traditional masculinity ideology when considering the role of men in sexual relationships and reproductive health. Our findings suggest gender transformative policies and programs seeking to inspire men for more gender-equitable relationships with their partners.

## 1. Introduction

In the 1994 International Conference on Population and Development and the Fourth World Conference on Women in 1995, the importance of gender relations was brought to the foreground as an important plan in the sexual and reproductive health (RH) discourses. The objective was to promote gender equality by encouraging men to get involved in matters of sexual and RH; for example, using contraceptives and preventing unwanted pregnancies and sexually transmitted diseases (1).

Most studies on sexual and RH have focused mainly on women, while the attitude of men is rarely considered. This article and the proposed hypotheses are motivated by findings from previous studies that have emphasized on the importance of examining the relationship between gendered attitude and sexual and RH (2, 3). In Malaysia, much of the research has employed education and sexual health perspective in order to analyze people's knowledge of sexual and RH issues (4–7). However, little attention has been paid to gender perspective in order to examine men's attitude toward sexual relationships (SRs) and RH. Filling this gap in the literature marks a significant theoretical contribution of this study to masculinity and sexual and RH literature in Malaysia.

Moreover, this study utilizes partial least squares-structural equation modelling (PLS-SEM), an advanced analysis technique, to assess the influence of men's masculine attitude and behavior on gender norms related to SRs and RH. In addition to using PLS-SEM to assess the model proposed for this study, applying new and locally developed scales – KANITA Masculinity Scale (KMS) (8) and localized Gender-Equitable Men (GEM) Scale (9) – can be regarded as a methodological contribution of the current study.

In brief, this article aims to investigate how Malaysian men's masculine gender-role attitude and traditional behavior influence SRs and RH.

## 2. Materials and Methods

This cross-sectional study was undertaken by administrating the localized GEM scale to measure attitude toward SRs and RH among Malaysian men. A questionnaire survey was administered to 168 men living in urban areas in Peninsular Malaysia between April and May 2016. The questionnaires were administered in Bahasa Malay language.

The inclusion criteria of the study were: men who were currently sexually active, living in urban areas in Peninsular Malaysia and aged between 20 and 64 yr with different levels of education but with adequate proficiency in Bahasa Malaysia. Men who were not sexually active and those who were living in rural areas were excluded.

Using the 10-times rule (10) and the threshold of 100 sample size, a sample of 168 respondents was enrolled for PLS-SEM analysis (11).

### Instrument development and measures 

#### GEM

To assess men's attitude toward gender norms related to SRs and RH, we applied the localized version of the GEM scale (9), with 12 items identifying inequitable subscale and 9 items for equitable subscale. The types of items in this scale included Violence, SRs, Domestic chores and daily life, and RH with three response categories including “disagree,” “partially agree,” and “agree.” Indeed, higher scores indicate greater support for inequitable gender norms.

#### KMS

To measure Malaysian men's masculine gender-role attitude and behavior, the KMS was used, a psychometrically validated masculinity scale in Malaysian context developed by the Centre for Research on Women and Gender (KANITA) in Universiti Sains Malaysia (8). KMS is a multidimensional scale with a total of 68 items divided into four subscales, namely physical attributes (7 items); personality traits (28 items), behavior (17 items) in two components labelled as “traditional behaviour” and “non-traditional behavior,” and gender roles (16 items) in three components labelled as “men's position of control” (MPC) representing the nature of men as the leading one in society and family, “men's responses to family” (MRF) representing men's traditional responsibilities toward his family, and “feminine roles in men” (FRM) representing responsibilities that were not traditionally taken up by men. The KMS is scored on a seven-point Likert scale with (1) “disagree,” (3) “neutral,” and (7) “very much agree.” For the current study, we used “traditional behaviors” and all three components of “gender roles” items of KMS (8).

Before data collection, a pilot test with 100 male respondents was conducted to assess the questionnaire reliability and validity. The Cronbach's alpha value for equitable and inequitable subscales in Malaysian version of the GEM scale were 0.83 and 0.80, respectively. The Cronbach's alpha values for equitable and inequitable subscales in Malaysian version of the GEM scale, “traditional behavior” and all components reported in “gender roles” subscales were well above the value of 0.80 (8). Thus, it was indicated that KMS and GEM are reliable and valid to be used to facilitate studies related to masculinity and gender norms in Malaysia.

### Ethical considerations

This research was approved by the Human Research Ethics Committee of Universiti Sains Malaysia (USM/JEPeM/14090323). Respondents were approached from different districts, ages, and races and assured of the confidentiality of the results. A written consent form was obtained from the men who were willing to participate. They were confident that participation was completely voluntary and had no effect on their routine care. If the men consented, they were given the questionnaires to complete.

### Statistical analysis

Data were analyzed using SmartPLS v. 3.2.6 (SmartPLS GmbH, Germany), a software for variance-based structural equation modelling (SEM) using the partial least squares (PLS) path modelling method. The proposed conceptual model was examined by assessing the measurement and structural models. In assessing measurement model, composite reliability (CR) and average variance extracted (AVE) were tested. The bootstrapping test was used to calculate the *t*-statistics and to test the significance of estimated path coefficients in structural model. The statistical significance of path coefficients, Cronbach's alpha, HTMT, and *R*² values were tested by using Bootstrapping method (12).

## 3. Results

Table I shows the profile of the respondents categorized into five age groups: 20–29 (40.5%), 30–39 (25.0%), 40–49 (22.0%), 50–59 (10.7%), and ≥ 60 yr (1.8%). The majority of respondents were Malay (61.3%) followed by Chinese (30.4%) and Indian (7.7%). Of the 168 samples, a small percentage (4.8%) had only primary- or secondary-level education. Approximately half of the respondents (49.4%) had a high school certificate or diploma. Moreover, 45.3% had graduated from a university or institution of higher education. Among the 168 respondents, 93 (55.4%) were married, 61 (36.3%) were single, while a small number were either engaged (5.4%) or divorced (3.0%).

### Model assessment using PLS–SEM

#### Assessment of the measurement model 

This study investigates six reﬂective variables, namely, men's traditional behaviour (MTB); men's response to family (MRF); FRM; MPC; RH; and SR. Composite reliability (CR), indicator reliability, average variance extracted (AVE), and discriminant validity were evaluated to assess the measurement model (10) (Table II). The criterion for internal consistency reliability in PLS-SEM is CR, which refers to the different outer loadings of each construct, as values between 0.7 and 0.95 can be considered as satisfactory. For indicator reliability, items with the loadings ≥ 0.7 are acceptable. However, weaker outer loadings might be observed in social science studies, especially when newly developed scales are applied (13). As reported in Table II, each item on its associated construct had a loading ≥ 0.6, and both the CR and AVE for the constructs were ≥ 0.7 and ≥ 0.5, respectively, thus representing acceptable reliability. Owing AVEs ≥ 0.5 also shows that each of the constructs had an acceptable convergent validity, demonstrating that on average, the construct explains more than half of the variance of its indicators.

Discriminant validity refers to the extent to which a construct or variable is unique and captures a distinct phenomenon rather than other constructs in the model (10). To assess the discriminant validity, two criteria were evaluated: Fornell-Larker and HTMT ratio. The results of both ratios indicate the model proposed in this study possesses acceptable discriminant validity.

#### Assessment of the structural model

To assess the structural model, two criteria including the value of the R${}^{2}$ coefficients as well as the significance of the path coefficients should be evaluated and reported. The *t*-values were used to check the level of significance of the path coefficients. Based on the behavioral research standards, the R${}^{2}$ value of 0.2 is generally considered high (10). In this study, the value of R${}^{2}$ for RH and SR were 0.133 and 0.137, respectively; showing relatively acceptable R${}^{2}$ values and that MTB, MRF, MPC, and FRM are relatively good predictors of RH and SR.

**Figure 1:**
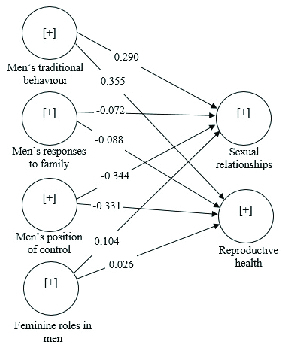
Results of assessment of structural model.

Table III and Figure 1 show the results of the structural model. The results of this study support the positive and significant effects of MTB and MPC on RH (H1, H5) and SR (H2, H6). The findings indicate that the effect of MPC on SR (H6) is much stronger than that of MTB (H2). The findings, however, do not support the effects of MRF and FRM on RH (H3, H7) and SR (H4, H8).

**Table 1:** Profile of respondents


**Characteristics **		**Frequency (%)**
**Age (yr)**		
	**20–29**	68 (40.5%)
	**30–39**	42 (25.0%)
	**40–49**	37 (22.0%)
	**50–59**	18 (10.7%)
	**≥ 60**	3 (1.8%)
**Level of education**		
	**Primary and secondary school**	8 (4.8%)
	**High school (grade 11/12)**	83 (49.4%)
	**Higher education (Degree/Master/Ph.D.)**	76 (45.3%)
	**Other**	1 (0.6%)
**Ethnicity**		
	**Malay**	103 (61.3%)
	**Chinese**	51 (30.4%)
	**Indian**	13 (7.7%)
	**Other**	1 (0.6%)
**Marital status**		
	**Single**	61 (36.3%)
	**Engaged **	9 (5.4%)
	**Married**	93 (55.4%)
	**Divorced**	5 (3.0%)

**Table 2:** Assessment results of the measurement model


**Construct/Item**		**Loading**	**CR**	**AVE**
**Men's traditional behavior**				
	**Hygienic**	0.827	0.920	0.625
	**Good at managing time**	0.914		
	**Practical**	0.906		
	**Discipline**	0.776			
	**Can control own emotion**	0.745		
	**Focus on own career**	0.638*		
	**Diplomatic**	0.687*		
**Men's response to family**				
	**I protect my family**	0.707	0.877	0.642
	**I fulfill the needs of my family**	0.841		
	**I make the final decision in my family**	0.873		
	**I work to support my family**	0.775		
**Feminine roles in men**				
	**I cook for my family**	0.800	0.870	0.691
	**I do chores at home**	0.782		
	**I send and pick up my children to/ from school**	0.907		
**Men's position of control**				
	**I was paid higher than women in my family and friends**	0.749	0.887	0.567
	**I am a better leader than women around me**	0.787		
	**I rank higher than women in my family and friends**	0.760		
	**I am eligible to be the head of state**	0.681*		
	**I am eligible to be a religious leader**	0.770		
	**I deserve to be the head of the family**	0.766		
**Reproductive health**				
	**Men should be outraged if their wives ask them to use a condom**	0.914	0.817	0.692
	**Women who carry condoms on them are easy**	0.741		
**Sexual relationship**				
	**It is the man who decides what type of sex to have**	0.747	0.817	0.530
	**Men need sex more than women do**	0.618*		
	**You don't talk about sex; you just do it**	0.739		
	**Men are always ready to have sex**	0.795		
Loadings show indicator reliability, AVE or “Average variance extracted” indicates convergent validity, CR or “Composite reliability” indicate internal consistency, all calculated with PLS Algorithm. *Loading between 0.4 and 0.7 can be considered acceptable if CR and AVE are ≥ 0.7 and ≥ 0.5, respectively				

**Table 3:** Results of hypothesis testing


	**Hypothesis**	**Path coefficient**	*t* -value*	**Supported**
**H1**	MTB→RH	0.355	2.196	Yes
**H2**	MTB→SR	0.290	1.838	Yes
**H3**	MRF→RH	–0.088	0.833	No
**H4**	MRF→SR	–0.072	0.497	No
**H5**	MPC→RH	0.331	4.133	Yes
**H6**	MPC→SR	0.344	3.750	Yes
**H7**	FRM→RH	0.026	0.239	No
**H8**	FRM→SR	0.104	0.984	No
Hypothesis tests based on PLS-SEM, a multivariate statistical technique combining factor analysis and multiple regression analysis, *Significant at p < 0.05 if *t* ≥ 1. 46 (one-tailed), calculated with bootstrapping procedure (5,000 samples)				

## 4. Discussion

This study is one of the first gender studies using the localized GEM scale and newly developed masculinity scale to investigate gendered attitude of Malaysian men. The analysis using PLS-SEM reveals new evidence regarding the influence of masculine gender role attitude and behavior on SRs and RH.

Using the framework of masculinity ideology, previous studies have indicated that men's masculine gender-role attitude and traditional behavior have negative effects on the quality of their intimate relationships, sexual activities, and RH (14). Masculinity ideology, as a conceptualization of the male gender role, refers to men's beliefs about how men are expected to behave and adhere to culturally defined standards for male gender roles (15). As discussed by Ward and colleagues, “traditional masculinity ideology focuses on the power of the male sex drive, on men as sexual agents and initiators, and on women as sexual objects” (16). Men's acceptance of these ideologies will result in inequality in SRs and RH matters. The findings of our study confirm the arguments. The findings show that MTB and MPC are positively associated with inequality in SRs (H2, H6) and RH (H1, H5). Men who offer stronger endorsement of traditional behaviors and MPC over women report more negative condom attitude and greater priority of men's desires in SRs. As discussed by Morokoff, in such context where men traditionally are in the position of control over women, “a woman is expected to facilitate a man's sexual needs, relieve sexual tension within the relationship, and focus on the man's pleasure, rather than hers” (17). In other words, within the context of masculine culture and traditional norms, men are sexually seen as active and women as passive (18). These differing roles are commonly known as the “sexual double standard” that gives men more sexual rights to initiate sexual activity and decide when and what type of sex to have, while, in turn, induce women to feel responsible for men's sexual desires and needs (19); all representing inequality in SRs.

Our findings are consistent with the results from previous studies that indicate relationships between masculine gender roles and inequality in RH particularly the condom use. Gender inequality in RH and contraceptive use can be identified through the mechanism in which men usually see contraception as something that women should take care of and men do not consider it essential for themselves to be involved in this area, particularly in condom use that is perceived as reducing sexual pleasure (20). Walcott and his collaborators found that men who strictly abide by higher masculine gender norms orientations and traditional behavior showed less tendency to take the initiative or a serious action toward preventing unplanned pregnancies (21). Studies conducted in Iran show that perceived masculine gender roles were the main factors affecting gender inequality in SRs and RH particularly men's reluctance in using condoms, joint sexual decision-making, and male pleasure predominating in sexual encounters (20, 22).

Considering the aforementioned discussion, the results of the current study are consistent with the masculinity ideology framework and previous studies, and consequently confirm the influence of MPC and traditional behavior on inequality in SRs and RH.

This study had a limitation of generalizability caused by the fact that the sample was drawn from Malaysia as a multi-ethnic country. Comparative studies between different ethnic groups with different cultures might elucidate these findings and advance our understanding of the factors influencing SRs and RH.

## 5. Conclusion

Our findings highlight the importance of taking into account the traditional masculinity ideology when considering the role of men in SRs and RH. Given the influence of men's masculine gender role attitude and traditional behavior on SRs and RH, our findings suggest gender transformative policies and programs seeking to inspire men for more gender-equitable relationships with their partners.

##  Conflict of Interest

None declared.

## References

[B1] United Nations http://www.un.org/popin/icpd2.htm.

[B2] Lefkowitz ES, Shearer CL, Gillen MM, Espinosa-Hernandez G (2014). How gendered attitudes relate to women’s and men’s sexual behaviors and beliefs. Sex Cult.

[B3] Santana MCh, Raj A, Decker MR, La Marche A, Silverman JG (2006). Masculine gender roles associated with increased sexual risk and intimate partner violence perpetration among young adult men. J Urban Heal.

[B4] Awang H, Wong LP, Jani R, Low WY (2014). Knowledge of sexually transmitted diseases and sexual behaviours among Malaysian male youths. J Biosoc Sci.

[B5] Ismail SB, Muhamad R, Hussain NHN, Leon P

[B6] Ling JES, Tong SF (2017). The roles of men in family planning: A study of married men at the UKM primary care clinic. Malays Fam Physician.

[B7] Low WY (2004). Impact of sexual health course on Malaysian university students. Med J Malaysia.

[B8] Centre for Research on Women and Gender (KANITA)

[B9] Sukumaran V, Sc The validation and application of bahasa Malaysia gender equitable men (GEM) Scale: An assessment of equitable and inequitable gender norms among male student of a Malaysian University [M. thesis].

[B10] Hair JF, Hult GTM, Ringle Ch, Sarstedt M (2017). A primer on partial least squares structural equation modeling (PLS-SEM).

[B11] Reinartz W, Haenlein M, Henseler J (2009). An empirical comparison of the efficacy of covariance-based and variance-based SEM. Int J Res Mark.

[B12] Ringle CM, Wende S, Becker JM (2015). “SmartPLS 3.

[B13] Hulland J (1999). Use of partial least squares (PLS) in strategic management research: A review of four recent studies. Strateg Manag J.

[B14] Noar SM, Morokoff PJ (2002). The relationship between masculinity ideology, condom attitudes, and condom use stage of change: A structural equation modeling approach. Int J Mens Health.

[B15] Pleck J, Sonenstein F, Ku L (2010). Masculinity ideology: Its impact on adolescent males’ heterosexual relationships. J Soc Issues.

[B16] Ward LM, Merriwether A, Caruthers A (2006). Breasts are for men: Media, masculinity ideologies, and men’s beliefs about women’s bodies. Sex Roles.

[B17] Morokoff PJ, Travis CB, White JW A cultural context for sexual assertiveness in women. Sexuality, society, and feminism.

[B18] Ippolito JM, D Women’s sexuality, assertiveness, and relationship satisfaction [Ph. Thesis], Department of Professional Psychology.

[B19] Impett EA, Peplau LA (2002). Why some women consent to unwanted sex with a dating partner: Insights from attachment theory. Psychol Women Q.

[B20] Fallahi H, Tavafian SS, Yaghmaiee F, Hajizadeh I (2012). Perceived barriers of condom use in people living with HIV/AIDS: A qualitative research. ] Payesh.

[B21] Walcott MM, Ehiri J, Kempf MC, Funkhouser E, Bakhoya M, Aung M, et al (2015). Gender norms and family planning practices among men in western jamaica. Am J Mens Health.

[B22] Lotfi R, Ramezani-Tehrani F, Merghati-Khoei E, Yaghmaei F, Dworkin ShL

